# Parental Bacillus Calmette-Guérin vaccine scars decrease infant mortality in the first six weeks of life: A retrospective cohort study

**DOI:** 10.1016/j.eclinm.2021.101049

**Published:** 2021-08-12

**Authors:** MLT Berendsen, F. Schaltz-Buchholzer, P. Bles, S. Biering-Sørensen, KJ. Jensen, I. Monteiro, I. Silva, P. Aaby, CS. Benn

**Affiliations:** a Bandim Health Project, Department of Clinical Research, University of Southern Denmark and Odense University Hospital, Odense, Denmark; b Bandim Health Project, Indepth Network, Bissau, Guinea-Bissau; c Department of Internal Medicine, Radboud Center for Infectious Diseases, Radboud University Medical Center, Nijmegen, the Netherlands; d Experimental and Translational Immunology, Department of Health Technology, Technical University of Denmark, Kgs Lyngby, Denmark; e Center for Clinical Research and Prevention, Frederiksberg Hospital, Frederiksberg, Denmark; f Danish Institute for Advanced Study, University of Southern Denmark, Denmark

**Keywords:** Bacille Calmette-Guérin, Non-specific effects of vaccines, Heterologous effects, Parental priming, Vertical boosting, Immunological inheritance

## Abstract

**Background:**

Live attenuated vaccines have been observed to have particularly beneficial effects for child survival when given in the presence of maternally transferred immunity (priming). We aimed to test this finding and furthermore explore the role of paternal priming.

**Methods:**

In an exploratory, retrospective cohort study in 2017, parental Bacillus Calmette-Guérin (BCG) scars were assessed for infants from the Bandim Health Project (BHP) who had participated in a 2008–2013 trial of neonatal BCG vaccination. Parental scar effects on mortality were estimated from birth to 42 days, the age of the scheduled diphtheria-tetanus-pertussis (DTP) vaccination, in Cox proportional hazard models adjusted with Inverse Probability of Treatment Weighting.

**Findings:**

For 66% (510/772) of main trial infants that were still registered in the BHP area, at least one parent was located. BCG scar prevalence was 77% (353/461) among mothers and 63% (137/219) among fathers. In the first six weeks of life, maternal scars were associated with a mortality reduction of 60% (95%CI, 4% to 83%) and paternal scars with 49% (-68% to 84%). The maternal scar association was most beneficial among infants that had received BCG vaccination at birth (73% (-1% to 93%)). Although priming was less evident for paternal scars, having two parents with scars reduced mortality by 89% (13% to 99%) compared with either one or none of the parents having a scar.

**Interpretation:**

Parental BCG scars were associated with strongly increased early-life survival. These findings underline the importance of future studies into the subject of inherited non-specific immunity and parental priming.

**Funding:**

Danish National Research Foundation; European Research Council; Novo Nordisk Foundation; University of Southern Denmark.

**Research in context**

**Evidence before this study**

We searched PubMed without restrictions to English language publication using the search string: (BCG [tiab] OR Bacillus Calmette-Guerin [tiab]) AND (maternal [tiab] OR paternal [tiab]) AND (non-specific OR nonspecific OR heterologous). BCG and other live-attenuated vaccines are associated with reduced overall mortality and morbidity among children. Maternal BCG vaccination/scarification increases this beneficial effect among their BCG-vaccinated offspring, possibly due to a ‘trained immunity’ phenotype with increased pro-inflammatory cytokine production. No studies on paternal BCG vaccination were found, nor studies on non-BCG-vaccinated infants.

**Added value of this study**

Both maternal and paternal BCG scars are associated with improved early-life survival of their offspring. For maternal scar, associations were especially beneficial among those children who received BCG at birth. This was less apparent for paternal scar, but having a second parent with a BCG scar greatly improved associations with survival.

**Implications of all the available evidence**

Our findings highlight the need for studies on inherited non-specific immunity, including paternal effects. The studies on parental BCG vaccination indicate that its non-specific effects are not limited to the vaccinated individual, but could extent to their offspring. This makes universal BCG vaccination policies, as well as efforts to increase BCG scarring by revaccination of scar-negative individuals possibly low-cost interventions to improve health for current and future generations.

Alt-text: Unlabelled box

## 1 Introduction

Bacillus Calmette-Guérin (BCG) and other live attenuated vaccines have been associated with beneficial non-specific effects (NSEs); they increase child survival by protecting against unrelated infections [1], [2], [3], [4]. In a meta-analysis of three trials from 2017, BCG-at-birth vs. the usual delayed-BCG to low-weight (LW) infants reduced neonatal all-cause mortality by 38% (95% CI, 17% to 54%) and infant mortality by 16% (0% to 29%) [2]. Neonates that received early BCG also had reduced in-hospital mortality, especially due to fewer cases of fatal neonatal sepsis [5].

Interestingly, it has been shown that beneficial NSEs of live attenuated vaccines can be enhanced if the infant is primed by maternal immunity [6], [7], [8], [9]. This was first reported in a combined analysis of two RCTs on early measles vaccine (MV), where vaccination in presence of maternally-derived measles antibodies was associated with a 78% (36% to 93%) reduced mortality compared with vaccination in the absence of maternally-derived measles antibodies [6].

Subsequently, maternally primed BCG effects have been described in both low- and high-income settings. In Danish neonates, BCG vaccination reduced hospital admission due to infections by 35% (6% to 55%) when their mother had received BCG, whereas there was no effect in neonates born to BCG-unvaccinated mothers (p for interaction=0.01) [7]. In Bissau-Guinean infants of 4.5 months, having a BCG scar, a marker of a correctly applied BCG,[10] was associated with mortality reductions of 41% (5% to 64%). Noteworthy, this beneficial scar effect was only seen among infants whose mothers had a BCG scar (66% [33% to 83%] vs. 8% [−83% to 53%] with no maternal scar) [8].

To further explore the role of parental BCG priming and examine the potential interaction with infant BCG vaccination we took advantage of the most recent of three BCG trials,[2] where infants had been randomised to BCG-at-birth or the standard practice of delayed BCG (typically provided at around six weeks of age). We hypothesised that the combination of maternal BCG scar and BCG-at-birth would be associated with lower mortality from 0 to 42 days, when most of the control group infants had not yet received BCG and before all infants would receive other routine vaccines.

## 2 Methods

### 2.1 Setting

The Bandim Health Project (BHP) maintains a Health and Demographic Surveillance System (HDSS) in six urban districts in Bissau, Guinea-Bissau. Births are registered by BHP staff at the country's main hospital and during regular trimonthly home-visits. Routine vaccinations are provided at three HDSS health centres in accordance with the WHO-recommended immunization schedule.

### 2.2 Study design

**Fig. 1:**
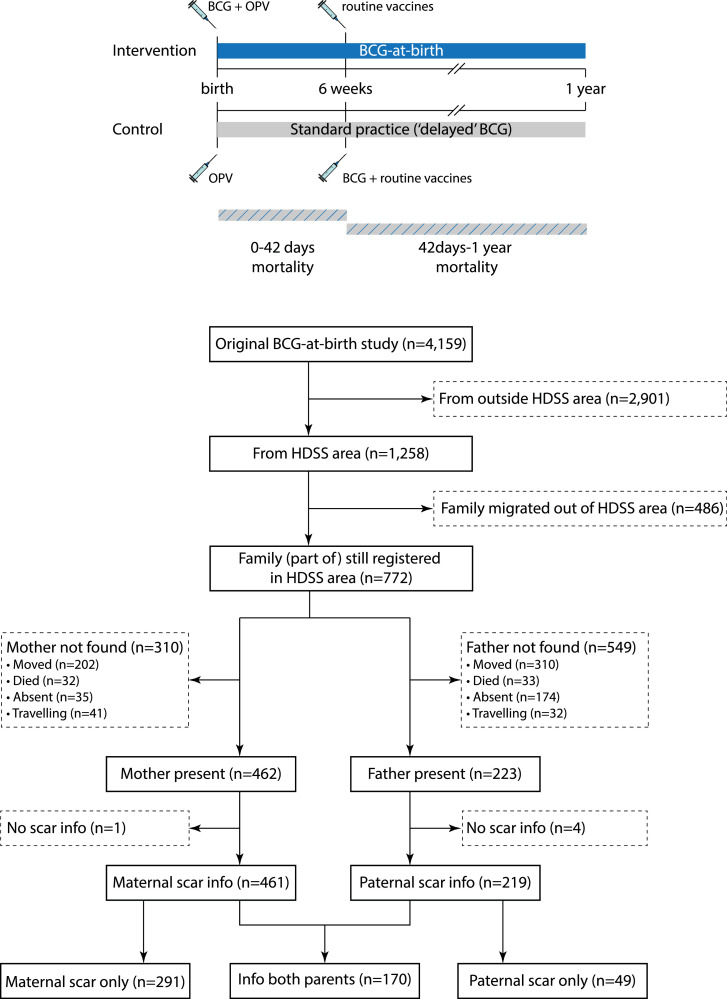
Study design of the original trial and flowchart of study participants. In the original trial infants were randomized to BCG-at-birth or standard practice (‘delayed’ BCG). The BCG vaccination at 6 weeks in the control group is an indication from when most children in the control group would start receiving BCG, not the age to which the control group was randomized. Abbreviations: BCG, bacillus Calmette-Guérin; BHP, Bandim Health Project; HDSS, Health and Demographic Surveillance System.

The original RCT design has been described in detail previously [2]. Briefly, the trial was designed to test the effects of early BCG vaccination (intervention group) versus delayed BCG vaccination (control group, standard practice) on neonatal mortality among LW infants, with infant mortality as a secondary outcome. Between 2008 and 2013, 4159 infants weighing <2500 g were recruited at four hospitals and the three HDSS health centres. Infants were randomised (1:1) to 0·05 mL intradermal BCG-Denmark vaccine (Statens Serum Institut) (intervention group, ‘BCG-at-birth’) or standard practice; that is, mothers were encouraged to have their infant BCG vaccinated at the local health center when the infant had gained weight (control group, ‘delayed BCG’). All infants received oral polio vaccine (OPV) at inclusion and we conducted follow-up visits at three days after enrolment and at 2, 6, and 12 months of age (Fig. 1). The family of infants who died were visited three months after the death by a specially trained field assistant to conduct a WHO/INDEPTH verbal autopsy [11].

### 2.3 Study population and parental BCG scar assessment

During the original trial, which included 4159 infants from all parts of greater Bissau, we had not been aware of the possible interaction between maternal and infant BCG, and no parental BCG information had been collected. For the present retrospective cohort study, all infants from the original trial that had lived within the HDSS area at inclusion (*N* = 1258) were sought for in the HDSS system in February 2017. Families of infants who had not moved according to the latest census information (*N* = 722) were visited, including parents of infants who had died during the original trial. We did not perform a sample size calculation as it was impossible to estimate the proportion of parents that could be reached and would be willing to participate. We conducted up to three home visits to find both parents. Parents who were present, received an explanation about the present study being an extension of the original BCG trial. After informed oral consent, BCG scars were assessed and measured with a ruler by one of two field assistants. Field assistants were unaware of the hypothesis regarding parental BCG scars. We have previously shown that a BCG scar is an indicator for a correctly applied BCG vaccine[10,12,13] and its use, instead of reported BCG vaccination, overcomes possible problems with recall bias.

### 2.4 Outcomes

While the original trial reported effects of BCG on the all-cause neonatal mortality for historical reasons, our primary outcome was all-cause mortality for the period up to 42 days of age, before the infants would have reached the age where other routine vaccines are administered. We have done so in subsequent RCTs evaluating BCG versus no BCG (NCT02504203) and RCTs evaluating effects of different BCG strains (NCT03400878 and NCT04383925). The scheduled diphtheria-tetanus-pertussis (DTP) vaccination at six weeks of life has been associated with negative effects on overall female survival and might distort the effects of early BCG [14,15]. Furthermore, the majority of the infants in the delayed BCG group will not have received BCG by this age. Secondary outcome was all-cause mortality between 42 days and 1 year of age.

### 2.5 Statistical analyses

Infant mortality rates for 0–42 days and 42 days-1 year were compared according to maternal and paternal scar status in Cox proportional hazard models, providing mortality rate ratios (MRRs). Two children (1 with both maternal and paternal scar, 1 only paternal scar) were enrolled in the trial later than 42 days after birth and were thus only included in the 42 day-1 year analysis. Time from randomization to all cause death was our main outcome and survival time was censored at migration out of HDSS area, or end of follow-up (42 days or 1 year). Clustering of twins was adjusted for by using robust standard errors. The proportional-hazards assumption was assessed graphically and tested using Schoenfeld residuals. In case there were no deaths in one of the groups, a univariate log-rank test was performed.

Models were adjusted using stabilised Inversed Probability of Treatment Weighting (sIPTW). Probability of the infant's parent to have a BCG scar was predicted from a logistic regression including age, maternal schooling, electricity, and indoor toilet based on a directed acyclic graph (DAG) for this study (Supplementary Figure 1). Weights were stabilised using the marginal probability for exposure in the numerator. The assessment of correctly weighted samples is summarised in Supplementary Table 1 [16]. Effect modification by BCG randomization and sex was determined by including an interaction term to the analyses.

Sensitivity analyses for outcome were performed using not 0–42day mortality, but neonatal mortality as the outcome, as well as censoring infants in the delayed BCG group at the day of BCG receipt. Sensitivity analyses for statistical adjustment included using different models for the prediction of the probability of exposure for sIPTW; 1) addition of place of inclusion and weight at inclusion, 2) addition of the subgroup variable (BCG randomization/sex), 3) prediction within the subgroups. The latter two analyses were to ensure sIPTW did not bias estimates due to possible different effects within subgroups, as detailed by Izem and colleagues [17].

Statistical analyses were performed using Stata 12 (Stata Corp, College Station, Texas).

### 2.6 Ethics

The original protocol was approved by the Guinean Ministry of Health's Research Coordination Committee and consultative approval was obtained from the Danish Central Ethical Committee and contained a statement on participants being approached for future studies. The parental scar follow-up amendment was approved by the same instances. Both the parental scar follow-up (NCT03020147) and the original study (NCT00625482) were registered at clinicaltrials.gov. The manuscript adheres to the STROBE guidelines.

### 2.7 Role of the funding source

The funder of the study had no role in study design, data collection, data analysis, data interpretation, or writing of the report. All authors had full access to all the data in the study and had final responsibility for the decision to submit for publication.

## 3 Results

The original BCG trial included 1258 infants from the BHP HDSS (Fig. 1). At the start of the parental BCG scar screening, 772 infants had not migrated out of the HDSS. Of these, 66% (510/772) had at least one parent (mother: 462, father 223) present in the household at a visit. Most of the parents that were not present, had moved after the latest census (mother: 202/310, father: 310/549). Characteristics of included and excluded infants are described in Supplementary Table 2.

BCG scar information was obtained for 100% (461/462) of the mothers that were present and 98% (219/223) of the fathers, with a scar prevalence of 77% (353/461) and 63% (137/219), respectively. In the group of 170 infants that had information for both parents’ scar status, BCG scar prevalence was 76% (130/170) among mothers and 62% (105/170) among fathers. The baseline characteristics for infants did not differ with respect to their parents’ BCG scar status, except for twin status (Table 1 and Supplementary Table 3). Twins were more common among mothers with a BCG scar (21% (73/353) vs. 7% (8/108)), while they were less common among fathers with a BCG scar (15% (21/137) vs. 33% (27/82)). In addition, infants of mothers with a scar were more likely to have been enrolled at a hospital and at an earlier age in the original trial, and their mothers had more years of schooling (Table 1).

**Table 1:** Baseline characteristics by maternal and paternal BCG scar status.

	Infants,% (No./Total)^a^			Infants,% (No./Total)^a^		
	Maternal BCG scar *N* = 353	No maternal BCG scar *N* = 108	P value^c^	Paternal BCG scar *N* = 137	No paternal BCG scar *N* = 82	P value^c^
Male sex	37 (130/353)	37 (40/108)	0.97	34 (47/137)	35 (29/82)	0.87
Age at randomization, days	2 (1–7)	3.5 (1–10)	**0.04**	2 (1–7)	4 (1–9)	0.12
Allocation to early BCG	53 (186/353)	53 (57/108)	0.99	48 (66/137)	54 (44/82)	0.43
Included in hospital	65 (230/353)	47 (51/108)	**0.001**	63 (86/137)	61 (50/82)	0.79
Twin/triplet	21 (73/353)	7 (8/108)	**0.002**	15 (21/137)	33 (27/82)	**0.002**
Caesarian section^b^	5 (17/352)	5 (4/107)	0.95	4 (5/137)	6 (5/80)	0.38
Premature^b^	24 (53/222)	15 (7/47)	0.18	20 (17/86)	29 (13/45)	0.24
Mother alive at enrollment	–	–	–	100 (137/137)	100 (82/82)	–
Maternal MUAC, mm^b^	246 (232–268)	248 (232–262)	0.55	247 (232–265)	248 (236–270)	0.77
Maternal age, years^b^	29 (24–35)	33 (25–38)	**0.03**	30 (25–34)	33 (28–38)	**0.005**
Paternal age, years^b^	39 (33–47)	42 (37–48)	0.13	36 (30–46)	43 (38–49)	**<0.001**
Anthropometrics						
Weight at inclusion, kg	2.24 (2.02–2.39)	2.27 (2.05–2.40)	0.22	2.23 (1.99–2.39)	2.21 (1.91–2.36)	0.28
Length, cm^b^	45.9 (44.0–47.0)	46.0 (44.0–47.0)	0.43	45.5 (44.0–46.5)	45.0 (43.5–47.0)	0.56
Head circumference, cm	32.0 (31.0–33.0)	32.0 (30.5–32.8)	0.88	31.5 (30.5–32.5)	31.9 (30.5–33.0)	0.77
Abdominal circumference, cm	27.0 (26.0–28.1)	27.4 (26.0–28.5)	0.51	27.1 (26.0–28.2)	27.0 (26.0–28.4)	0.87
MUAC, mm	82 (76–88)	82 (76–88)	0.96	82 (76–88)	82 (76–88)	0.86
Socio-economics						
Maternal schooling, years	7 (3–9)	4 (0–8)	**<0.001**	6 (2–9)	5 (0–9)	0.11
Electricity in the house	39 (138/353)	34 (37/108)	0.37	41 (56/137)	35 (29/82)	0.46
Indoor toilet	22 (76/353)	21 (23/108)	0.96	23 (32/137)	20 (16/82)	0.51
Zinc roof^b^	97 (342/353)	99 (107/108)	0.31	99 (134/136)	98 (80/82)	0.63

a Data represent the% (No./Total) of infants for categorical variables and median (25p-75p) for continuous variables.

b Different n due to missings, which are <10% except for maturity (131, 61, 51 and 37 missing), as this was only measured when child was born in the national hospital.

c P values calculated with Chi-square or Fisher's exact test for categorical variables and Wilcoxon ranksum test for continuous variables. Abbreviations: BCG, Bacillus Calmette-Guérin; MUAC, mid-upper arm circumference.

### 3.1 Effect of parental BCG scars in the first 6 weeks of life

**Fig. 2:**
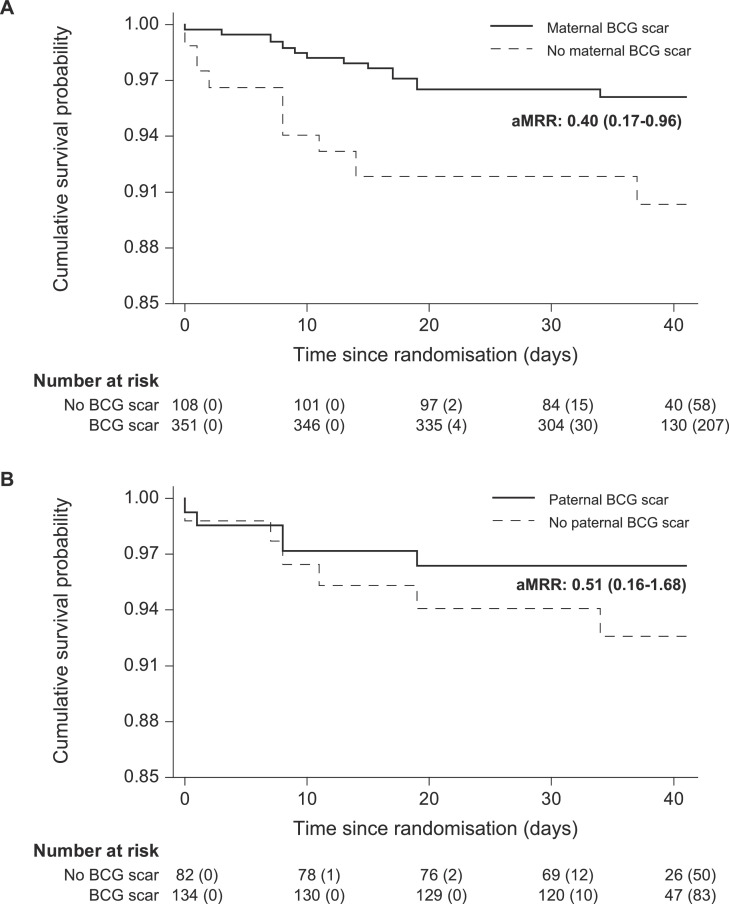
Kaplan-Meier curve of survival probability by parental BCG scar status. Adjusted mortality rate ratios (aMRRs) for maternal BCG scar (A) and paternal BCG scar (B) estimated in a Cox proportional hazards model with time since randomization as the underlying time variable. Models were adjusted by stabilized Inverse Probability of Treatment Weighting (sIPTW) based on the variables maternal schooling, electricity, indoor toilet and age of the child at inclusion. Observations were censored at migration or 42 days of age, whatever came first. Numbers at risk and numbers censored (bracketed) are rounded to the nearest integer because of the sIPTW. Abbreviations: BCG, Bacillus Calmette-Guérin.

Two infants were recruited after six weeks of age, leaving 459 infants with maternal scar information among which there had been 22 (13 scar vs. 9 no scar) deaths in the first six weeks of life. Among the 217 infants with paternal scar information, there had been 11 deaths (5 scar vs. 6 no scar). This resulted in crude MRRs of 0·42 (0·18 to 0·98) for maternal scar and 0·49 (0·15 to 1·60) for paternal scar (Table 2). After adjustment, the aMRRs were 0·40 (0·17 to 0·96) and 0·51 (0·16 to 1·68), respectively (Fig. 2). The association of a parent's scar with survival was enhanced by the presence of a scar in the other parent (Table 3). Maternal scar aMRR was 0·04 (0·00 to 0·32) in the presence of a paternal scar and paternal scar aMRR was 0·13 (0·01 to 1·18) in the presence of a maternal scar. When both parents had a scar, the aMRR was 0·11 (0·01 to 0·87) compared with either one or none of the parents having a scar (Table 3).

**Table 2:** Effect of maternal and paternal BCG scars on mortality between birth and 42 days.

	Mortality rate [Deaths/1000 Person days] (n)		MRR (CI) (Scar/No scar)	Adjusted MRR (CI) (Scar/No scar)^b^
	BCG scar	No BCG scar		
Maternal^a^	1.0 [13/12.6] (352)	2.6 [9/3.5] (107)	**0.42 (0.18–0.98)**	**0.40 (0.17–0.96)**^c^
Paternal^a^	1.0 [5/4.8] (135)	2.2 [6/2.7] (82)	0.49 (0.15–1.60)	0.51 (0.16–1.68)^d^

a Two infants included after 42 days (1 both maternal and paternal scar, 1 only paternal scar).

b The Cox proportional hazards model was adjusted by stabilized Inverse Probability of Treatment Weighting based on the variables maternal schooling, electricity, indoor toilet and age of the child at inclusion.

c Proportional hazards = 0.54. ^d^Proportional hazards = 0.29. Abbreviations: BCG, Bacillus Calmette-Guérin; CI, confidence interval; MRR, mortality rate ratio.

**Table 3:** Effect of parental BCG scarring on mortality between birth and 42 days for children with scar information available for both parents.

	Mortality rate [Deaths/1000 Person days] (n)		MRR (CI) (Paternal scar/No paternal scar)	Adjusted MRR (CI) (Paternal scar/No paternal scar)^b^
	Paternal BCG scar^a^	No paternal BCG scar^a^		
Maternal BCG scar^a^	0.32 [1/3.2] (87)	2.8 [4/1.4] (42)	0.12 (0.01–1.03)	0.13 (0.01–1.18)^e^
No maternal BCG scar^a^	6.2 [3/0.5] (16)	2.6 [2/0.8] (23)	2.38 (0.41–13.74)	2.80 (0.49–16.05)^f^
MRR (CI) (Maternal scar/No maternal scar)	**0.06 (0.01–0.50)**	1.09 (0.20–5.88)		
Adjusted MRR (CI) (Maternal scar/No maternal scar)^b^	**0.04 (0.00–0.32)**^c^	1.14 (0.21–6.16)^d^		**0.11 (0.01–0.87)**^g^

a Paternal scar data is missing for 291 infants of whom we have maternal scar data, maternal scar data is missing for 49 of whom we have paternal scar data, and two infants were included after 42 days, leaving 168 infants with information of both parents.

b Separate Cox proportional hazards models were performed for maternal and paternal scar status, which were adjusted by stabilized Inverse Probability of Treatment Weighting based on the variables maternal schooling, electricity, indoor toilet and age of the child at inclusion.

c Proportional hazards = 0.10.

d Proportional hazards = 0.58.

e Proportional hazards = 0.79.

f Proportional hazards = 0.12.

g aMRR estimate for children with both parents having a scar compared to children with either one or none of their parents having a scar, proportional hazards = 0.41. Abbreviations: BCG, Bacillus Calmette-Guérin; CI, confidence interval; MRR, mortality rate ratio.

### 3.2 Interaction between parental BCG scar and infant characteristics

The main trial had randomised infants to early BCG or standard practice (delayed BCG). In the delayed BCG group (*N* = 239), where most infants had not yet received BCG at 42 days (191/239), both maternal scar (aMRR: 0·49 (0·15 to 1·63)) and paternal scar (aMRR: 0·41 (0·09 to 1·83)) were associated with a more than halved mortality, although not significant (Table 4). In the early BCG group (*N* = 271), the association between maternal scar and mortality was especially beneficial (aMRR: 0·27 (0·07 to 1·01)) (Table 4), while this was not seen for paternal scar. Hence, receiving BCG-at-birth was most beneficial when the mother had a BCG scar (aMRR: 0·37 (0·11 to 1·20)). Interaction with infant's sex revealed differential effects of parental BCG scars with more beneficial associations for same-sex parent-infant pairs (Supplementary Table 4).

**Table 4:** Effect of maternal and paternal scar on mortality between birth and 42 days, by BCG vaccination allocation.

	Mortality rate [Deaths/1000 Person days] (n)		Adjusted MRR (CI) (Early BCG/Delayed BCG)^a^
	Early BCG	Delayed BCG	
Maternal BCG scar	0.60 [4/6.6] (185)	1.5 [9/5.9] (167)	0.37 (0.11–1.20)^b^
No maternal BCG scar	2.7 [5/1.9] (57)	2.4 [4/1.6] (50)	0.67 (0.17–2.56)^b^
Paternal BCG scar	0.85 [2/2.3] (65)	1.2 [3/2.5] (70)	0.72 (0.12–4.26)^c^
No paternal BCG scar	1.4 [2/1.5] (44)	3.2 [4/1.3] (38)	0.43 (0.08–2.27)^c^
Adjusted MRR (CI) (Maternal scar/No maternal scar)^a^	0.27 (0.07–1.01)^b^	0.49 (0.15–1.63)^b^	
Adjusted MRR (CI) (Paternal scar/No paternal scar)^a^	0.69 (0.10–4.77)^c^	0.41 (0.09–1.83)^c^	

a Separate Cox proportional hazards models were performed for infants with maternal and paternal scar information, which contained an interaction with allocation group and were adjusted by stabilized Inverse Probability of Treatment Weighting based on the variables maternal schooling, electricity, indoor toilet and age of the child at inclusion.

b P for interaction = 0.51, proportional hazards = 0.23.

c P for interaction = 0.67, proportional hazards = 0.44. Abbreviations: BCG, Bacillus Calmette-Guérin; CI, confidence interval; MRR, mortality rate ratio.

### 3.3 Sensitivity analyses

Parental BCG scar associations remained unchanged after conducting the analysis for the neonatal period (up to 28 days), or censoring infants in the delayed BCG group when they received BCG before six weeks of age (*N* = 48) (Supplementary Table 5). The associations were also robust to changes in statistical adjustment, whether this was done by addition of place of inclusion and weight at inclusion to the IPTW, by addition of the subgroup variable (randomization/infant's sex) to the IPTW or calculation of the IPTW per subgroup (Supplementary Table 5).

### 3.4 Effect of parental BCG scars from 6 weeks to 1 year of life

Between six weeks and 1 year of age, there were no beneficial associations between parental BCG scars and infant survival (Table 5). In contrast to 0–42 days, where a maternal scar was most beneficial in the early BCG group, the largest association for maternal scar was in the delayed BCG group, where most infants had received BCG at or after six weeks of age (aMRR: 0·38 [0·08 to 1·73]) (Supplementary Table 6). The same aMRR for paternal scar was 0·71 (0·10 to 5·07).

**Table 5:** Effect of maternal and paternal BCG scars on mortality between 42 days and 1 year of age.

	Mortality rate [Deaths/100 Person years] (n)		MRR (CI) (Scar/No scar)	Adjusted MRR (CI) (Scar/No scar)^c^
	BCG scar	No BCG scar		
Maternal	4.8 [14/2.9] (340)	4.7 [4/0.86] (99)	1.03 (0.34–3.10)	1.60 (0.51–5.05)^d^
Paternal^a^	4.4 [5/1.1] (132)	3.1 [2/0.65] (75)	1.41 (0.27–7.35)	1.80 (0.34–9.43)^e^
Both parents^b^	4.0 [3/0.75] (87)	3.1 [2/0.64] (73)	1.27 (0.21–7.57)	1.53 (0.26–9.20)^f^

a One infant migrated within 42 days (no paternal scar).

b BCG scar’ equals both parents with a scar and ‘No BCG scar’ equals either one or none of the parents with a scar.

c The Cox proportional hazards model was adjusted by stabilized Inverse Probability of Treatment Weighting based on the variables maternal schooling, electricity, indoor toilet and age of the child at inclusion.

d Proportional hazards = 0.48.

e Proportional hazards = 0.14.

f Proportional hazards = 0.59. Abbreviations: BCG, Bacillus Calmette-Guérin; CI, confidence interval; MRR, mortality rate ratio.

## 4 Discussion

This is the first study to evaluate the effects on early-life mortality of paternal and maternal BCG scars. We report mortality reductions in the first six weeks of life of 60% (4% to 83%) for maternal scars and of 49% (−68% to 84%) for paternal scars. These parental scar effects were enhanced when both parents had a scar. Between six weeks and 1 year of life, beneficial effects of parental BCG scar were absent. The study corroborated our hypothesis that the combination of maternal BCG scar and BCG-at-birth is associated with lower mortality. This combined effect was less evident for paternal scar.

The retrospective study design influenced the power of our study. Although we included families who had moved within the study area at their new address, we could not assess the scars of parents who were absent or had migrated. Due to the lack of ‘addresses’ outside the study area and the facts that social media was not as omnipresent during the original trial and mobile phone numbers change rapidly these families could not be traced. Included infants were more often male, had mothers with higher mid-upper arm circumferences and more often had electricity at home, but sampling bias from the original study appears unlikely as the mortality rates in the first six weeks of life were similar between included and excluded infants: 4·5% (23/508) vs. 4·3% (32/746) (Supplementary Table 2). However, sampling bias cannot be ruled out completely, since the sampling of the original study might not have generated a representative sample.

Comparison of early-life mortality by parental BCG scar status is subject to factors that could have had an impact on the probability of developing a BCG scar for the parents. Parents were also more likely to have had concordant scar status (110) than discordant scar status (58). There was, however, little socioeconomic difference between parents with and without a scar (Supplementary Table 3). The only difference between parents with and without a scar was age. Older parents were less likely to have a BCG scar, presumably reflecting improved BCG vaccination since it became general policy in the late 1980s as correct vaccination technique is one of the main determinants for developing a BCG scar [12,13]. Parental age in itself did not determine child survival in the first six weeks of life.

The use of a DAG and IPTW allowed us to correct for several potential confounders despite the small number of events. Residual confounding cannot be ruled out, but our results were robust to changes in the IPTW with respect to including additional variables that either differed between the groups or greatly influenced the outcome. Furthermore, the estimates were robust to subgroup effects and limiting the analysis to the neonatal period.

Due to the limited number of events, we could not conduct interaction analyses using combined parental scar data or examine combined effects of randomization and sex. In addition, several analyses had low power, especially for the effects of paternal scars and in the post-six weeks period. Hence, most analyses are explorative and should be interpreted with caution. Nevertheless, it should be noted that the hypothesis for maternal BCG scar and mortality was based on previous observations [6,7].

Studies on live attenuated vaccines have demonstrated that vaccination in the presence of pre-existing immunity, acquired via previous vaccination or maternally transferred immunity, increases their beneficial non-specific effects [1,7,8,[18], [19], [20]]. Our findings are in line with previous maternal BCG priming findings[7]^,^ [8] as maternal BCG scars were associated with enhanced survival between birth and 42 days in the early BCG group. After 42 days this effect was no longer seen which could indicate a short duration of the effect. Another possibility is an interaction with DTP vaccination given at 6 weeks of life. While subsequent DTP vaccination in the early BCG group is associated with potential deleterious effects on survival,[14] this is not found for simultaneous DTP vaccination which would have occurred in the delayed BCG group [21].

The immune mechanisms underlying NSEs of BCG are not fully understood and most likely feature both heterologous innate and T-cell responses of which the former are termed ‘trained immunity’ and are most widely described for the NSEs of BCG [22], [23], [24]. Ugandan infants born to mothers with a BCG scar showed enhanced cytokine production in cord blood compared with infants from mothers without BCG scars [25]. Also after infant BCG vaccination, gene expression profiles indicated increased pro-inflammatory immune function among infants whose mothers had a BCG scar. In BCG-vaccinated Australian infants the effect of maternal BCG scar was more balanced [26]. Infants of mothers with a BCG scar had higher responses of interleukin-1β to BCG and interferon gamma (IFN-γ) to *Streptococcus pneumoniae*, but lower responses of monokine induced by IFN-γ to *Haemophilus influenzae* and macrophage inflammatory protein 1α to Pam3CSK4 and peptidoglycan [26]. Immunological studies incorporating paternal influences are still to be done.

Trained immunity after vaccination is probably mediated by epigenetic modifications, such as DNA methylation, miRNAs or histone modifications, [27] although genetic variation might play a role as well [28]. Histone modifications have been shown to be present in circulating NK-cells and monocytes after BCG vaccination [22,29]. Inheritance of epigenetic profiles from parents to their offspring could be a potential mechanism for parental BCG priming effects. Epigenetic inheritance has been described for several species: systemic acquired resistance in plants, [30] longevity in fungi [31], and various traits in rodents [32]. In humans, studies are scarce, but there are suggestions for epigenetic inheritance via chromatin changes in spermatozoids [33]. Interestingly, some epigenetic traits show preferred sex-differential phenotypes, with inheritance towards offspring of the same sex [34,35]. This is similar to our sex-differential findings, but it should be mentioned that this analysis had low power and we cannot rule out combined effects of randomization and sex.

Our study corroborate several initial studies, indicating that the combination of maternal BCG priming and infant BCG vaccination reduces offspring mortality and hospital admissions in low- and high-income settings, respectively [7,8]. Therefore, since maternal priming might determine the NSEs of BCG vaccination, maternal BCG coverage should be taken into consideration when performing studies on the NSEs of BCG and might be a potential reason that BCG has shown differential effects in different studies [2,7]. If generalisable, it would be detrimental to ignore these effects by discontinuing BCG in the vaccination schedule, especially in countries where most mothers are still BCG immunised, such as Portugal and Ireland that only recently stopped BCG vaccination to new-borns.

In addition to the benefits of maternal priming, this study is the first to describe possible beneficial effects of paternal BCG scars. The paternal BCG scar estimate on early-life survival was similar to that of maternal scars. Although the interaction with infant BCG vaccination was less evident, having a father with a BCG scar amplified the beneficial association of a maternal scar on mortality and vice versa. In BCG unvaccinated infants, parental scar effects were underpowered. However, both paternal and maternal scars were associated with large reductions in early-life mortality. Since infants often receive delayed BCG vaccinations,[36] ensuring a high parental BCG scar prevalence might provide an important early-life health benefit which could have major implications for public health. For example, it should be assessed whether it is worthwhile to provide BCG vaccination to all women and/or men in the fertile age who do not have a BCG scar. To validate the current findings, an RCT among women in their fertile age randomized to BCG vaccination or control with follow-up of their offspring would be necessary. We are currently planning such a study, but due to its long duration observational studies in different settings could already strengthen the found results. As BCG boosting has been associated with beneficial non-specific benefits,[19] it might also be examined whether BCG revaccination of young adults entail a health benefit, both for themselves and future offspring. Immunological studies should include paternal influences and could target epigenetic changes in spermatozoids of BCG-vaccinated males.

In conclusion, both maternal and paternal BCG vaccine scars were associated with decreased mortality in the first six weeks of life, the effect being enhanced when both parents had a scar. While both parental scars were associated with reduced mortality among BCG unvaccinated infants, interaction with infant BCG vaccination was more evident for maternal BCG scars. The possibility of inherited health benefits of BCG underlines the importance of a general BCG vaccination policy and the need for future studies into the subject of inherited non-specific immunity.

## Author contributors

CSB and PA conceived and designed both the original BCG trial and the parental BCG scar study. SBS, KJJ and FSB supervised the field data collection of the original trial. MLTB, PB and IS supervised the additional field data collection conducted for the parental BCG scar study. IM was responsible for the data collection and data entry within the original trial and the parental BCG scar study. MLTB and PB verified the underlying data and MLTB performed all statistical analyses. FSB and IS assisted in conceptualizing the manuscript and MLTB wrote the first draft of the manuscript. All authors contributed to the final version of the manuscript.

## Declaration of Competing Interest

MLTB and FSB received support from the University of Southern Denmark in the form of a scholarship. CSB holds an ERC starting grant and PA holds a research professorship from Novo Nordisk. PB, SBS, KJJ, IM, and IS declare no conflict of interest.

## Appendix Supplementary materials

Image, application 1
