# Supplementary material for: Parental Bacillus Calmette-Guérin vaccine scars decrease infant mortality in the first six weeks of life: A retrospective cohort study
Source: eClinicalMedicine. 2021 Aug 12;39:101049. doi: 10.1016/j.eclinm.2021.101049 (PMC8365433; doi:10.1016/j.eclinm.2021.101049)
Supplement: Supplementary file 1 [file mmc1.docx]

**SUPPLEMENTARY MATERIAL**

**Supplementary Figure 1.** Directed acyclic graph for effects of parental BCG scarring on all-cause mortality between birth and 42 days

**Supplementary Table 1.** Assessment of stabilized Inverse Probability of Treatment Weights

**Supplementary Table 2.** Baseline characteristics of children in the original trial, stratified by whether they could be nor could not be included in the present study

**Supplementary Table 3.** Baseline characteristics of children with maternal and paternal BCG scar information, by scar status

**Supplementary Table 4.** Sensitivity analyses for the effect of maternal and paternal BCG scars on mortality between birth and 42 days

**Supplementary Table 5.** Effect of maternal and paternal scar on mortality between birth and 42 days, by sex of the infant

**Supplementary Table 6.** Effect of maternal and paternal scar on mortality between 42 days and 1 year of age, by BCG vaccination allocation

**
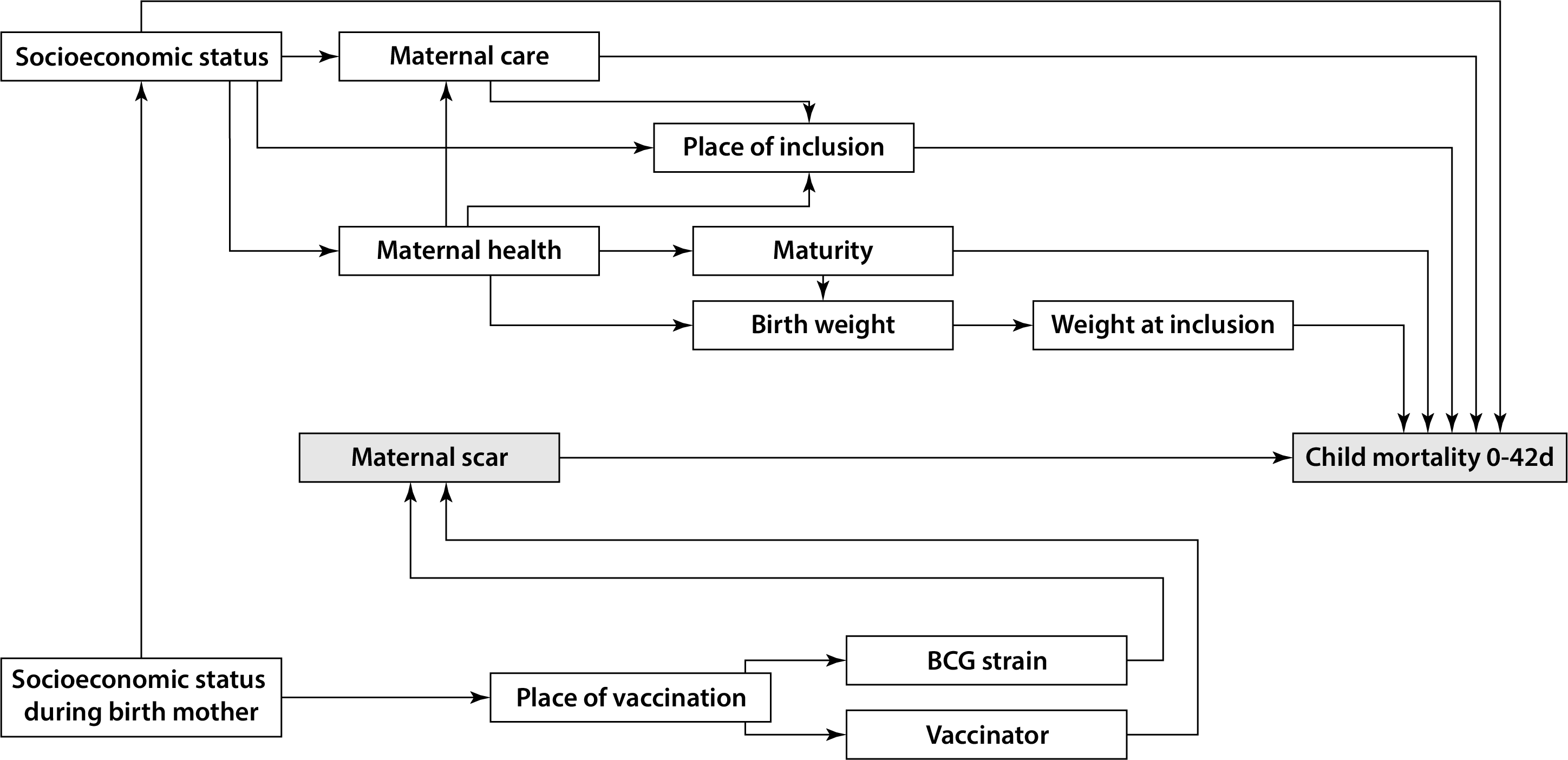
**

**Supplementary Figure 1. Directed acyclic graph for effects of parental BCG scarring on all-cause mortality between 0-42 days.** Abbreviations: BCG, Bacillus Calmette-Guérin.

**Supplementary Table 1. Assessment of stabilized Inverse Probability of Treatment Weights**

|  | Probability receiving treatment | Stabilized weights | Standardized difference | | | | | | | |
| --- | --- | --- | --- | --- | --- | --- | --- | --- | --- | --- |
|  |  |  | Maternal schooling | | Age | | Electricity | | Indoor toilet | |
|  |  |  | Before  sIPTW | After  sIPTW | Before  sIPTW | After  sIPTW | Before  sIPTW | After  sIPTW | Before  sIPTW | After  sIPTW |
| Maternal BCG scar |  |  |  |  |  |  |  |  |  |  |
| Birth - 42 days | 0.77 ±0.09 (0.40-0.89) | 1.001 ±0.215 (0.40-2.11) | 42.3% | -1.5% | -26.4% | 0.9% | 8.2% | 2.3% | 0.46% | -0.7% |
| 42 days -1year | 0.77 ±0.09 (0.36-0.90) | 1.001 ±0.233 (0.36-2.24) | 44.0% | -2.3% | -30.0% | 1.2% | 9.9% | 3.1% | -1.8% | -0.8% |
|  |  |  |  |  |  |  |  |  |  |  |
| Paternal BCG scar |  |  |  |  |  |  |  |  |  |  |
| Birth - 42 days | 0.63 ±0.06 (0.45-0.74) | 1.000 ±0.130 (0.77-1.42) | 22.2% | -1.2% | -13.2% | -0.2% | 9.2% | -1.0% | 7.6% | -1.9% |
| 42 days -1year | 0.63 ±0.05 (0.40-0.73) | 1.001 ±0.120 (0.74-1.59) | 16.7% | -1.5% | -15.5% | 0.0% | 12.6% | -0.9% | 10.9% | -2.0% |
|  |  |  |  |  |  |  |  |  |  |  |
| Both parents scar |  |  |  |  |  |  |  |  |  |  |
| Birth - 42 days | 0.52 ±0.05 (0.39-0.61) | 1.000 ±0.104 (0.82-1.33) | 18.2% | -0.2% | -6.2% | -2.6% | -2.9% | 0.2% | -0.9% | -0.1% |
| 42 days -1year | 0.54 ±0.07 (0.34-0.67) | 1.000 ±0.139 (0.77-1.59) | 19.6% | -0.6% | -9.3% | 1.4% | -10.1% | 0.7% | -3.9% | -0.2% |
| Values are depicted as mean ±standard deviation (range) or as percentages. Probabilities of receiving treatment were received from logistic regressions with scar presence as the dependent variable and maternal schooling, electricity, indoor toilet and age of the child at inclusion as covariates. Standardized weights were calculated as (marginal probability of treatment) / (probability of receiving treatment from logistic regression) for children whose parent had a scar and (1 - marginal probability of treatment) / (1 - probability of receiving treatment from logistic regression) for children whose parent had no scar. Standardized differences were calculated as 100 * (meanscar – meannoscar) / square root((varscar + varnoscar) / 2) for continuous variables and as 100 * (probscar – probnoscar) / square root((probscar * (1 – probscar) + probnoscar * (1 – probnoscar)) / 2) for dichotomous variables. Abbreviations: BCG, Bacillus Calmette-Guérin; CI, confidence interval; MRR, mortality rate ratio. Abbreviations: sIPTW, stabilized Inverse Probability of Treatment Weighting. | | | | | | | | | | |

**Supplementary Table 2. Baseline characteristics of children in the original trial, stratified by whether they could be nor could not be included in the present study**

|  | Infants^a^ | |  |
| --- | --- | --- | --- |
|  | Included  N=510 | Excluded  N=748 | P value^d^ |
| Male sex | 36 (185/510) | 27 (205/748) | **0.001** |
| Age at randomization, days | 3 (1-8) | 2 (1-7) | 0.64 |
| Allocation to early BCG | 47 (239/510) | 49 (370/748) | 0.36 |
| Included in hospital | 62 (317/510) | 67 (498/747) | 0.10 |
| Twin/triplet | 19 (95/510) | 16 (117/748) | 0.17 |
| Caesarian section^b^ | 5 (25/508) | 6 (42/746) | 0.58 |
| Premature^b^ | 23 (71/305) | 28 (344/479) | 0.13 |
| Mother alive at enrollment | 100 (510/510) | 100 (741/741) | - |
| Maternal MUAC, mm^b^ | 247 (232-266) | 244 (226-262) | **0.02** |
| Anthropometrics |  |  |  |
| Weight at inclusion, kg | 2.24 (2.00-2.39) | 2.24 (1.95-2.37) | 0.20 |
| Length, cm^b^ | 45.8 (44.0-47.0) | 45.5 (44.0-47.0) | 0.09 |
| Head circumference, cm | 32.0 (30.5-33.0) | 31.9 (30.5-32.8) | 0.17 |
| Abdominal circumference, cm | 27.0 (26.0-28.3) | 27.0 (26.0-28.5) | 0.73 |
| MUAC, mm | 82 (76-88) | 82 (76-88) | 0.28 |
| Socio-economics |  |  |  |
| Maternal schooling, years^b^ | 6 (0-9) | 6 (0-9) | 0.39 |
| Electricity in the house^b^ | 37 (189/510) | 18 (209/742) | **0.001** |
| Indoor toilet^b^ | 21 (108/510) | 19 (143/741) | 0.42 |
| Zinc roof^b^ | 97 (496/509) | 96 (715/742) | 0.28 |
| Mortality between birth and 42 days^c^ | 4.5 (23/508) | 4.3 (32/746) | 0.84 |
| ^a^Data represent the % (No./Total) of infants for categorical variables and median (25p-75p) for continuous variables. ^b^Different n due to missings, which are <10% except for maturity (205 and 269 missing), as this was only measured when child was born in the national hospital. ^c^Two children were included after 42 days in both groups. ^d^P values calculated with Chi-square or Fisher’s exact test for categorical variables and Wilcoxon ranksum test for continuous variables. Abbreviations: BCG, Bacillus Calmette-Guérin; MUAC, mid-upper arm circumference. | | | |

**Supplementary Table 3. Baseline characteristics of children with maternal and paternal BCG scar information, by scar status**

|  | Infants^a^ | |  |
| --- | --- | --- | --- |
|  | Both parents with BCG scar  N=88 | Only one or no parents with BCG scar  N=82 | P value^c^ |
| Male sex | 39 (34/88) | 33 (27/82) | 0.44 |
| Age at randomization, days | 3 (1-7.5) | 2.5 (1-8) | 0.82 |
| Allocation to early BCG | 49 (43/88) | 48 (39/82) | 0.87 |
| Included in hospital | 63 (55/88) | 55 (45/82) | 0.31 |
| Twin/triplet | 18 (16/88) | 22 (18/82) | 0.54 |
| Caesarian section^b^ | 3 (3/88) | 5 (4/80) | 0.71 |
| Premature^b^ | 22 (12/55) | 18 (7/40) | 0.60 |
| Mother alive at enrollment | 100 (88/88) | 100 (82/82) | - |
| Maternal MUAC, mm^b^ | 244 (232-268) | 248 (238-270) | 0.44 |
| Maternal age, years | 30 (25-34) | 33 (28-38) | **0.02** |
| Paternal age, years^b^ | 37 (31-45) | 43 (37-49) | **<0.001** |
| Anthropometrics |  |  |  |
| Weight at inclusion, kg | 2.22 (1.98-2.39) | 2.26 (2.07-2.38) | 0.64 |
| Length, cm^b^ | 45.6 (43.6-46.5) | 45.7 (44.0-47.0) | 0.37 |
| Head circumference, cm | 31.7 (30.5-32.5) | 32.0 (31.0-33.0) | 0.22 |
| Abdominal circumference, cm | 27.2 (26.0-28.0) | 27.5 (26.0-29.0) | 0.26 |
| MUAC, mm | 82 (76-88) | 88 (78-88) | 0.10 |
| Socio-economics |  |  |  |
| Maternal schooling, years^b^ | 6 (0-9) | 5 (0-9) | 0.24 |
| Electricity in the house | 41 (36/88) | 43 (35/82) | 0.82 |
| Indoor toilet | 23 (20/88) | 23 (19/82) | 0.95 |
| Zinc roof | 98 (86/88) | 99 (81/82) | 1.00 |
| ^a^Data represent the % (No./Total) of infants for categorical variables and median (25p-75p) for continuous variables. ^b^Different n due to missings, which are <10% except for maturity (33 and 42 missing), as this was only measured when child was born in the national hospital. ^c^P values calculated with Chi-square or Fisher’s exact test for categorical variables and Wilcoxon ranksum test for continuous variables. Abbreviations: BCG, Bacillus Calmette-Guérin; MUAC, mid-upper arm circumference. | | | |

**Supplementary Table 4. Sensitivity analyses for the effect of maternal and paternal BCG scars on mortality between birth and 42 days**

|  |  |  |  | Subgroup analysis | | | |
| --- | --- | --- | --- | --- | --- | --- | --- |
|  | Censoring delayed BCG group^a^ | Neonatal mortality | sIPTW with extra variables | Randomization | | Sex of the infant | |
|  |  |  |  | Main effect subgroup | sIPTW per subgroup | Main effect subgroup | Full effect subgroup |
| Maternal |  |  |  |  |  |  |  |
| Overall | **0.39 (0.16-0.93)** | **0.38 (0.15-0.95)** | **0.40 (0.17-0.98)** | **0.40 (0.17-0.96)** | **0.40 (0.17-0.96)** | **0.40 (0.17-0.95)** | **0.35 (0.15-0.86)** |
| Early BCG | 0.27 (0.07-1.01) |  |  | 0.27 (0.07-1.01) | **0.27 (0.07-0.99)** |  |  |
| Delayed BCG | 0.47 (0.14-1.55) |  |  | 0.49 (0.15-1.63) | 0.53 (0.16-1.67) |  |  |
| Males |  |  |  |  |  | 0.80 (0.20-3.26) | 0.70 (0.17-2.79) |
| Females |  |  |  |  |  | **0.27 (0.09-0.81)** | **0.25 (0.08-0.77)** |
|  |  |  |  |  |  |  |  |
| Paternal |  |  |  |  |  |  |  |
| Overall | 0.51 (0.16-1.68) | 0.59 (0.17-2.05) | 0.50 (0.14-1.78) | 0.49 (0.15-1.62) | 0.50 (0.15-1.64) | 0.51 (0.16-1.68) | 0.49 (0.15-1.62) |
| Early BCG | 0.69 (0.10-4.77) |  |  | 0.68 (0.10-4.74) | 0.59 (0.09-4.05) |  |  |
| Delayed BCG | 0.40 (0.09-1.82) |  |  | 0.41 (0.09-1.83) | 0.42 (0.09-1.94) |  |  |
| Males |  |  |  |  |  | -^b^ | -^b^ |
| Females |  |  |  |  |  | 1.42 (0.27-7.38) | 1.39 (0.26-7.27) |
|  |  |  |  |  |  |  |  |
| Both parents |  |  |  |  |  |  |  |
| Overall | **0.11 (0.01-0.87)** | **0.12 (0.02-0.96)** | **0.09 (0.01-0.67)** |  |  |  |  |
| Depicted are adjusted mortality rate ratios (95% Confidence intervals). The original Cox proportional hazards model from birth to 42 days, adjusted by stabilized Inverse Probability of Treatment Weighting (sIPTW) based on the variables maternal schooling, electricity, indoor toilet and age of the child at inclusion was altered in the following ways for the sensitivity analyses: 1) Censoring delayed BCG group: Infants were censored at the day of BCG receipt when this was within the first 6 weeks of life; 2) Neonatal mortality: mortality from birth to 28 days; 3) sIPTW with extra variables: sIPTW were based on the variables maternal schooling, electricity, indoor toilet, age of the child at inclusion, place of inclusion and weight at inclusion; 4) Main effect subgroup: sIPTW were based on the variables maternal schooling, electricity, indoor toilet, age of the child at inclusion and the subgroup variable (allocation/sex of the infant); 5) Full effect subgroup: sIPTW were based on the variables maternal schooling, electricity, indoor toilet, age of the child at inclusion within each arm of the subgroup variable (allocation/sex of the infant). ^a^Effect of paternal scar for boys was examined by Fisher’s exact test and therefore not suitable for the subgroup analyses. ^b^20% (48/239) of the children received BCG before 6 weeks of life in the delayed BCG group. Abbreviations: BCG, Bacillus Calmette-Guérin; sIPTW, stabilized Inverse Probability of Treatment Weighting. | | | | | | | |

**Supplementary Table 5.** **Effect of maternal and paternal scar on mortality between birth and 42 days, by sex of the infant**

|  | Mortality rate  [Deaths/1000 Person days] (n) | | Adjusted MRR (CI)  (Female/Male)^a^ |
| --- | --- | --- | --- |
|  | Female | Male |  |
| Maternal BCG scar | 0.88 [7/8.0] (223) | 1.3 [6/4.6] (129) | 0.66 (0.22-1.96)^b^ |
| No maternal BCG scar | 2.8 [6/2.2] (67) | 2.2 [3/1.4] (40) | 1.97 (0.47-8.20)^b^ |
| Paternal BCG scar | 1.6 [5/3.1] (89) | 0 [0/1.7] (46) | 0.10^c^ |
| No paternal BCG scar | 1.1 [2/1.8] (53) | 4.2 [4/1.0] (29) | 0.31 (0.06-1.65)^d^ |
| Adjusted MRR (CI)  (Maternal scar/No maternal scar)^a^ | **0.27 (0.09-0.81)^b^** | 0.80 (0.20-2.36)^b^ |  |
| Adjusted MRR (CI)  (Paternal scar/No paternal scar)^a^ | 1.41 (0.27-7.37)^d^ | **0.01^c^** |  |
| ^a^Separate Cox proportional hazards models were performed for infants with maternal and paternal scar information, which contained an interaction with sex of the infant and were adjusted by stabilized Inverse Probability of Treatment Weighting based on the variables maternal schooling, electricity, indoor toilet and age of the child at inclusion. ^b^P for interaction = .23, proportional hazards = .82. ^c^P values calculated with log-rank test due to no deaths among males in the paternal BCG scar group. ^d^Proportional hazards = .33. Abbreviations: BCG, Bacillus Calmette-Guérin; CI, confidence interval; MRR, mortality rate ratio. | | | |

**Supplementary Table 6.** **Effect of maternal and paternal scar on mortality between 42 days and 1 year of age, by BCG vaccination allocation**

|  | Mortality rate  [Deaths/100 Person years] (n) | | Adjusted MRR (CI)  (Early BCG/Delayed BCG)^a^ |
| --- | --- | --- | --- |
|  | Early BCG | Delayed BCG |  |
| Maternal BCG scar | 7.2 [11/1.5] (182) | 2.2 [3/1.4] (158) | 2.78 (0.78-9.98)^c^ |
| No maternal BCG scar | 0 [0/0.46] (52) | 10.1 [4/0.39] (47) | **0.03^b^** |
| Paternal BCG scar | 5.5 [3/0.55] (64) | 3.3 [2/0.60] (68) | 1.21 (0.20-7.13)^d^ |
| No paternal BCG scar | 0 [0/0.37] (42) | 7.2 [2/0.28] (33) | 0.11^b^ |
| Adjusted MRR (CI)  (Maternal scar/No maternal scar)^a^ | 0.07^b^ | 0.38 (0.08-1.73)^c^ |  |
| Adjusted MRR (CI)  (Paternal scar/No paternal scar)^a^ | 0.16^b^ | 0.71 (0.10-5.07)^d^ |  |
| ^a^Separate Cox proportional hazards models were performed for infants with maternal and paternal scar information, which contained an interaction with allocation group and were adjusted by stabilized Inverse Probability of Treatment Weighting based on the variables maternal schooling, electricity, indoor toilet and age of the child at inclusion. ^b^P values calculated with log-rank test due to no deaths among children in the early BCG group with no maternal BCG scar. ^c^Proportional hazards = .77. ^d^Proportional hazards = .21. Abbreviations: BCG, Bacillus Calmette-Guérin; CI, confidence interval; MRR, mortality rate ratio. | | | |
